# Multicentre Validation of the 2019 Briganti Nomogram: One Threshold Does Not Fit All

**DOI:** 10.1590/S1677-5538.IBJU.2026.0023

**Published:** 2026-04-30

**Authors:** Arthur Peyrottes, Fanny Orlhac, Alexandre Colau, Maxime Pattou, Yann Neuzillet, Fayek Taha, Stéphane Larré, François Desgrandchamps, Pierre Mongiat-Artus, Yves Allory, Alexandra Masson-Lecomte

**Affiliations:** 1 Université Paris Cité Hôpital Saint-Louis, AP-HP Department of Urology Paris France Department of Urology, Hôpital Saint-Louis, AP-HP, Université Paris Cité, Paris, France; 2 Laboratoire d’Imagerie Translationnelle en Oncologie Institut Curie Inserm U1288 Orsay France Laboratoire d’Imagerie Translationnelle en Oncologie, Inserm U1288, Institut Curie, Orsay, France; 3 Hôpital La Croix Saint-Simon Department of Urology Paris France Department of Urology, Hôpital La Croix Saint-Simon, Paris, France; 4 Université Versailles Saint-Quentin-en-Yvelines Hôpital Foch Department of Urology Suresnes France Department of Urology, Hôpital Foch, Université Versailles Saint-Quentin-en-Yvelines, Suresnes, France; 5 CHU de Reims Department of Urology Reims Franc Department of Urology, CHU de Reims, Reims, Franc; 6 Université Versailles Saint-Quentin-en-Yvelines Institut Curie Department of Pathology Saint-Cloud France Department of Pathology, Institut Curie, Université Versailles Saint-Quentin-en-Yvelines, Saint-Cloud, France

**Keywords:** Prostatic Neoplasms, Lymph Nodes, Nomograms

## Abstract

**Purpose:**

The 2019 Briganti nomogram is widely used to guide the indication for pelvic lymph node dissection (LND) at the time of radical prostatectomy in patients with localized prostate cancer. Although previously validated, its generalizability across distinct clinical settings remains uncertain.

**Materials and methods:**

We conducted a multicentre external validation of the nomogram in 481 patients from three French academic institutions (Centre A n=198, Centre B n=183 and Centre C n=100). Discrimination, calibration, and clinical utility were assessed. Spared LNDs and missed lymph node invasions (LNIs) were evaluated across risk thresholds.

**Results:**

The overall area under the receiver operating characteristics curve (AUC) was 0.733 but varied across centres (0.580-0.768). Calibration was acceptable overall but showed systematic overestimation in low-prevalence centres. At the 7% recommended threshold, the proportion of spared LNDs ranged from 51% to 76%, while missed LNIs ranged from 0% to 8.9%. Decision curve analysis revealed that the optimal threshold differed between centres.

**Conclusions:**

These results underscore the need for local validation and population-specific threshold adjustment before clinical implementation. Fixed thresholds may lead to under- or overtreatment depending on institutional case mix. Nomogram-based decision-making should be individualized based on local performance and patient-centred risk tolerance.

## INTRODUCTION

In contemporary urologic oncology, nomograms have become essential tools to support individualized clinical decision-making (1–3). By integrating multiple clinical, biological, and imaging variables, these models provide patient-specific risk estimates that often outperform traditional risk stratification systems. Their use has been associated with improved risk discrimination, better patient counselling, and more rational use of invasive procedures (4, 5). However, their performance is highly dependent on the population in which they are applied, and concerns remain regarding their transportability across different healthcare settings, particularly when derived from highly selected cohorts or single institutions.

In patients with localized prostate cancer (PCa), such nomograms have become integral to preoperative decision-making, particularly for evaluating the indication for pelvic lymph node dissection (LND) at the time of radical prostatectomy. The 2019 Briganti nomogram, developed by Gandaglia et al. (6), is one of the most widely adopted tools for predicting the risk of lymph node invasion (LNI), incorporating prostate-specific antigen (PSA), magnetic resonance imaging (MRI) findings, and results from both targeted and systematic biopsies. The authors proposed a 7% risk threshold above which an extended LND should be performed (6).

Although the 2019 Briganti nomogram underwent external validation shortly after its publication (7), its generalizability across diverse clinical settings remains incompletely characterized. We hypothesized that the nomogram's performance may vary depending on the population to which it is applied, thus requiring centre-specific validation. Furthermore, the optimal trade-off between the potential benefits and harms of LND likely depends on cohort-specific disease characteristics and may not align with the originally proposed 7% threshold. In the present study, we externally evaluated the 2019 Briganti nomogram in a multi-institutional cohort of patients diagnosed with PCa via MRI-targeted and systematic biopsies and investigated the optimal risk threshold for guiding the decision to perform extended LND.

## MATERIALS AND METHODS

After obtaining institutional review board (IRB) approval at each participating centre and for the overall cohort (IRB number: CRI-DATA DATA240274), we retrospectively identified 481 patients with clinically localized PCa who underwent robotic radical prostatectomy and extended pelvic lymph node dissection according to a standardized template including the external iliac, obturator, and hypogastric nodes (8). Patients were enrolled between October 2020 and December 2024 at three French academic institutions. All individuals were diagnosed using a combination of multiparametric magnetic resonance imaging (mpMRI)-targeted and concomitant systematic prostate biopsies. Magnetic resonance imaging was performed using either 1.5-T or 3.0-T scanners, following the European Society of Urogenital Radiology (ESUR) guidelines (9). The imaging protocol included T2-weighted, diffusion-weighted, and dynamic contrast-enhanced sequences. Lesions were scored according to the Prostate Imaging-Reporting and Data System (PI-RADS) version 2.1 (10) by uroradiologists with an experience in prostate MRI (>500 cases) at each centre. Targeted biopsies were conducted using elastic fusion with the KOELIS® system (KOELIS, La Tronche, France) by experienced urologists (>50 procedures a year for at least 5 years), with a minimum of two targeted cores sampled per index lesion. Systematic biopsies included a minimum of 6 to 12 cores outside the MRI index lesion. All prostatectomy specimens were evaluated by dedicated uropathologists at each centre following standardized histopathological protocols, in accordance with the 2019 International Society of Urological Pathology (ISUP) consensus (11). Pathological staging and grading were reported using the TNM system and ISUP grade group (11, 12), ensuring consistency across institutions. Patients were excluded if data on MRI, biopsy findings, or final pathology were incomplete.

The predicted probability of LNI for each patient was calculated using the logistic regression formula published in the original development study of the 2019 Briganti nomogram (6). The linear predictor (LP) was computed using the original coefficients (see supplementary Table-1). The predicted probability was then obtained by applying the logistic transformation: $p_{LNI}=\frac{1}{1+e^{-LP}}$ . Discrimination was evaluated using the area under the receiver operating characteristics (ROC) curve (AUC), and 95% confidence intervals (95% CI) were estimated via bootstrapping. Calibration was assessed using calibration plots, and clinical utility was assessed through decision curve analysis (DCA) across thresholds from 2% to 15%. Additionally, we calculated spared LNDs and missed LNIs at each threshold from 2% to 10%. The number of spared LNDs and missed LNIs was calculated by applying each risk threshold to the predicted probabilities: patients with predicted risks below the threshold were considered as candidates to omit LND (spared LNDs), and among these, those who had pathologically confirmed LNI were counted as missed LNIs. All statistical analyses were performed using R Studio Version 4.4.2 2024-10-31 (R Foundation for Statistical Computing, Vienna, Austria) employing the "pROC", "boot", "CalibrationCurves", and "dcurves" packages. This external validation study followed the TRIPOD (Transparent Reporting of a multivariable prediction model for Individual Prognosis Or Diagnosis) recommendations (13).

**Table 1:** Descriptives characteristic of the population for the whole population and for the three centres.

		Overall cohort (n=481)	Centre A (n=198)	Centre B (n=183)	Centre C (n=100)
**Age at surgery (yr), median (IQR)**		68 (62-73)	68 (63-72)	65 (61-75)	67 (61-70)
**Preoperative PSA (ng/mL), median (IQR)**		8.6 (5.93-12.7)	9.4 (6.57-14.8)	7.14 (5.57-10.4)	8.65 (5.53-17)
**Clinical stage at DRE, n (%)**					
	cT1	274 (57)	91 (46)	113 (61.5)	70 (70)
	cT2	201 (42)	102 (52)	69 (38)	30 (30)
	cT3	6 (1)	5 (2)	1 (0.5)	0 (0)
**PI-RADS score of index lesion, n (%)**					
	No lesion	20 (4)	3 (2)	3 (2)	14 (14)
	3	27 (6)	4 (2)	12 (6)	11 (11)
	4	234 (48)	90 (46)	96 (52)	48 (48)
	5	200 (42)	101 (50)	72 (40)	27 (27)
**Maximum lesion diameter of index lesion at MRI (mm), median (IQR)**		14 (10-20)	14.5 (11-20)	12 (10-17)	15 (10-20)
**ECE at MRI, n (%)**		69 (14)	30 (15)	30 (16)	9 (9)
**SVI at MRI, n (%)**		17 (4)	12 (6)	3 (2)	2 (2)
**ISUP grade group on targeted biopsy, n (%) Negative**		51 (11)	17 (9)	30 (16)	4 (4)
	1	93 (19)	16 (8)	66 (36)	11 (11)
	2	166 (35)	74 (37)	51 (28)	41 (41)
	3	77 (16)	40 (20)	17 (10)	20 (20)
	4	60 (12)	20 (10)	19 (10)	21 (21)
	5	34 (7)	31 (16)	0 (0)	3 (3)
**Number of cores taken at targeted biopsy, median (IQR)**		3 (2-4)	3 (2-4)	2 (2-3)	4 (3-6)
**Number of positive cores at targeted biopsy, median (IQR)**		2 (1-3)	2 (1-3)	2 (1-2)	4 (2-5)
**ISUP grade group on systematic biopsy, n (%)**					
	Negative	23 (5)	12 (6)	5 (3)	6 (6)
	1	36 (7)	6 (3)	22 (12)	8 (8)
	2	224 (47)	87 (44)	81 (44)	56 (56)
	3	131 (27)	67 (34)	40 (22)	24 (24)
	4	41 (9)	11 (6)	28 (15)	2 (2)
	5	26 (5)	15 (7)	7 (4)	4 (4)
**Number of cores taken at systematic biopsy, median (IQR)**		12 (12-12)	12 (6-12)	12 (12-12)	12 (10-15)
**Number of positive cores at systematic biopsy, median (IQR)**		4 (2-6)	4 (2-6)	4 (3-7)	3 (2-6)
**ISUP grade group (overall), n (%)**					
	1	12 (2)	1 (0.5)	7 (4)	4 (4)
	2	204 (42)	85 (43)	62 (34)	57 (7)
	3	156 (33)	75 (38)	49 (27)	32 (32)
	4	76 (16)	15 (7.5)	58 (31)	3 (3)
	5	33 (7)	22 (11)	7 (4)	4 (4)
**ISUP grade group at final pathology, n (%)**					
	1	32 (7)	1 (0.5)	30 (16)	1 (1)
	2	241 (50)	79 (40)	98 (54)	64 (64)
	3	151 (31)	85 (43)	36 (20)	30 (30)
	4	19 (4)	9 (4.5)	8 (4)	2 (2)
	5	38 (8)	24 (12)	11 (6)	3 (3)
**ECE at final pathology, n (%)**		190 (40)	95 (48)	54 (30)	41 (41)
**SVI at final pathology, n (%)**		52 (11)	32 (16)	14 (8)	6 (6)
**Positive surgical margin, n (%)**		165 (34)	67 (34)	70 (38)	28 (28)
**Number of lymph nodes removed, median (IQR)**		12 (9-15)	15 (12-18)	16 (10-20)	10 (8-12)
**Lymph node invasion at final pathology, n (%)**		38 (8)	28 (14)	8 (4)	2 (2)

Yr = Year; IQR = Inter-Quartile Range; DRE = Digital Rectal Examination; PI-RADS = Prostate Imaging – Reporting and Data System; MRI = Magnetic Resonance Imaging; ECE = Extra-Capsular Extension; SVI = Seminal Vesicle Invasion; ISUP = International Society of Urological Pathology.

## RESULTS

Population characteristics varied across centres (Table-1). Median PSA ranged from 7.14 to 9.4 ng/mL, and the proportion of extra-capsular extension and seminal vesicle invasion ranged from 9% to 15% and from 2% to 6% respectively. Prostate imaging reporting and data system (PIRADS) 5 lesions and International Society of Urological Pathology (ISUP) grade group 5 were more frequent in Centre A. The prevalence of LNI ranged from 2% (Centre C) to 14% (Centre A). Patients characteristics from the original "development" and "validation" cohorts can be found in Supplementary Table-2.

**Table 2:** Detailed threshold analysis. In blue is highlighted the proposed 7% threshold by Gandaglia et al. in their original study.

	Whole cohort (n=481)		Centre A (n=198)		Centre B (n=183)		Centre C (n=100)		2019 Briganti*	
	Spared LND	Missed LNI	Spared LND	Missed LNI	Spared LND	Missed LNI	Spared LND	Missed LNI	Spared LND	Missed LNI
**2%**	102 (21)	1 (1)	29 (15)	1 (3.4)	57 (31)	0 (0)	16 (16)	0 (0)	13 (3.2)	1 (7.7)
**3%**	214 (44)	4 (1.9)	66 (33)	2 (3)	109 (60)	2 (1.8)	39 (39)	0 (0)	164 (38)	2 (1.2)
**4%**	241 (50)	7 (2.9)	76 (38)	4 (5.3)	119 (65)	3 (2.5)	46 (46)	0 (0)	200 (47)	3 (1.5)
**5%**	249 (52)	8 (3.2)	79 (40)	4 (5.1)	123 (67)	4 (3.3)	47 (47)	0 (0)	217 (51)	4 (1.8)
**6%**	267 (56)	9 (3.4)	88 (44)	5 (5.7)	128 (70)	4 (3.1)	51 (51)	0 (0)	231 (54)	4 (1.7)
**7%**	296 (62)	13 (4.4)	101 (51)	9 (8.9)	139 (76)	4 (2.9)	56 (56)	0 (0)	244 (57)	4 (1.6)
**8%**	316 (66)	15 (4.7)	113 (57)	11 (9.7)	145 (79)	4 (2.8)	58 (58)	0 (0)	256 (60)	5 (2)
**9%**	334 (70)	16 (4.8)	118 (60)	11 (9.3)	153 (84)	5 (3.3)	63 (63)	0 (0)	266 (62)	6 (2.3)
**10%**	353 (73)	17 (4.8)	127 (64)	12 (9.4)	159 (87)	5 (3.1)	67 (67)	0 (0)	283 (66)	7 (2.5)

* Based on the original development study (Gandaglia et al. Eur Urol. 2019) (6). Data were not available for the external validation dataset (Gandaglia et al. Eur Urol. 2020).

**Figure 1:**
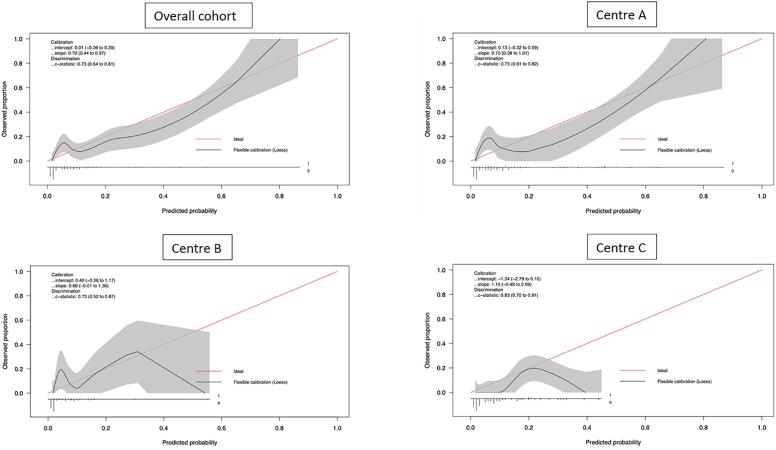
Calibration plots of predicted (ie, expected) versus observed probabilities of the populations.

Discrimination analysis showed variability in the nomogram's performance. The AUC values were 0.77 (95% CI 0.67-0.85) in Centre A, 0.73 (95% CI 0.43-0.91) in Centre B, and 0.58 (95% CI 0.37-0.86) in Centre C, with an overall AUC of 0.73 (95% CI 0.64-0.82). These were markedly lower than the 0.86 (95% CI not available) reported in the original development study (6) or the 0.79 (range 0.77-0.81) in the validation study (7). Calibration analysis showed reasonable alignment between predicted and observed risks overall, but miscalibration were obvious in Centre B and C, characterized by systematic overestimation of the predicted risk (Figure-1).

**Figure 2:**
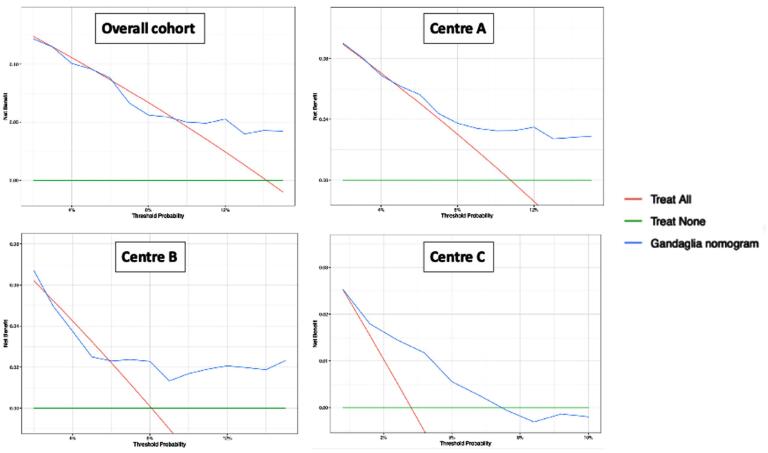
Decision-curve analysis assessing the net benefit associated with the use of the 2019 Briganti model for the detection of lymph node invasion at extended pelvic lymph node dissection depending on the cohort.

The number of spared LNDs and missed LNIs at different thresholds are summarized in Table-2. At the original 7% threshold, spared LNDs ranged from 51% (Centre A) to 56% (Centre C) and 76% (Centre B), while missed LNIs ranged from 0% (Centre C) to 2.9% (Centre B) and 8.9% (Centre A). Decision curve analyses (DCA) (Figure-2) revealed different optimal thresholds for each centre; the 7% threshold did not consistently yield the maximum net benefit.

## DISCUSSION

Our findings confirm the variable performance of the 2019 Briganti nomogram when applied across distinct clinical settings. While the overall discriminatory ability remained acceptable (AUC 0.73), significant heterogeneity was observed between centres, with AUC values ranging from 0.58 to 0.77. Calibration analysis further revealed systematic overestimation of risk in two of the three cohorts, and DCA demonstrated that the originally proposed 7% threshold did not consistently yield optimal net benefit. These results provide robust evidence that the nomogram, although previously externally validated, does not perform uniformly across populations and should not be applied in a one-size-fits-all manner.

First, the variability in nomogram performance highlights the inherent limitations of applying predictive tools developed in one setting to external populations. The original 2019 Briganti model demonstrated strong discrimination (AUC 0.86) in the development cohort (6) and a slightly reduced but still robust AUC (0.79) in its external validation (7). In contrast, our results show a broader range and, in some centres, suboptimal performance. These findings underscore the need for interpreting predictive performance metrics within the context of the population to which they are applied, particularly in light of differences in disease prevalence, risk distribution, and case mix (14).

Second, differences in patients and diseases’ characteristics between centres likely contributed to the observed heterogeneity. For instance, Centre A exhibited a higher prevalence of aggressive disease features such as PI-RADS 5 lesions and ISUP grade group 5, as well as a higher overall rate of LNI (14%) than Centres B and C. Conversely, Centre C had a very low LNI prevalence (2%), which likely limited the nomogram's discrimination and calibration in that setting. One could think that differences in LND templates would explain these results, but the median number of lymph nodes removed did not differ substantially between centres, supporting the homogeneity of the surgical template and suggesting that differences in nomogram performance were unlikely to be explained by variations in lymphadenectomy extent. These discrepancies may also reflect subtle variations in MRI interpretation, biopsy technique, or pathology review, even though all participating institutions followed standardized protocols. Importantly, the 2019 Briganti nomogram does not account for such inter-centre variability, which may undermine its generalizability. Hence, our data reinforce the need for local validation prior to clinical implementation of predictive models. Even when a model is robustly developed and externally validated, its utility in clinical decision-making depends on its performance within the target population. Discrimination, calibration, and decision-analytic utility should all be reassessed locally.

Third, our study provides concrete evidence that using a fixed decision threshold such as the 7% cutoff proposed by the original 2019 Briganti nomogram may lead to unintended consequences depending on the clinical context. For example, in Centre A, applying the 7% threshold would have spared 51% of patients from undergoing LND but at the cost of missing 8.9% of node-positive cases. In contrast, in Centre B, the same threshold would have spared 76% of LNDs while missing only 2.9% of LNIs, and in Centre C, it would have spared 56% of LNDs without missing any LNIs. These data illustrate the different trade-offs resulting from applying a uniform threshold across heterogeneous populations. Beyond the statistical measures of model performance, clinical decision-making ultimately depends on how much risk an individual urologist/patient is willing to accept to avoid harm. There may not be a single "correct" threshold, but rather a continuum of acceptable strategies informed by population data, clinical judgment and shared decision making with the patient. This highlights the importance of site-specific threshold selection based on both statistical evidence and real-world clinical considerations.

Taken together, these findings support the recommendation that each centre should validate the performance of nomograms within their own clinical environment. Where appropriate, thresholds should be adapted based on local data and applied within a shared decision-making framework. Nevertheless, the 2019 Briganti nomogram is of utmost importance and remains among the most accurate and extensively validated tools currently available for preoperative prediction of LNI in patients assessed with MRI-targeted and systematic biopsies (15, 16). Importantly, the field of preoperative risk stratification is evolving rapidly. Recent developments, including nomograms incorporating PSMA-PET imaging, show encouraging improvements in predictive accuracy (17). Notably, novel models developed by the same group have demonstrated enhanced diagnostic accuracy in patients staged with PSMA-PET/CT prior to surgery (18, 19).

Hence, our findings have direct implications for clinical practice. Rather than relying on fixed thresholds derived from external cohorts, clinicians should adopt a more dynamic approach integrating local data, institutional outcomes, and patient preferences. The use of decision curve analysis and centre-specific calibration may help refine the indication for lymph node dissection and reduce both overtreatment and undertreatment. Ultimately, embedding predictive models within a framework of shared decision-making and continuous local validation may represent a more sustainable strategy for precision surgery in prostate cancer.

These data should also be interpreted within the context of a rapidly evolving surgical ecosystem. The role of extended lymph node dissection itself remains debated, with emerging concepts of surgical de-escalation aiming to minimize morbidity without compromising oncological outcomes (20). However, recent high-level evidence has challenged this paradigm, suggesting a potential benefit of extended lymph node dissection on long-term oncological outcomes, including distant metastasis-free survival (21). In parallel, the increasing use of PSMA-PET imaging is reshaping preoperative staging and may lead to stage migration, potentially altering the performance of traditional nomograms developed in pre-PSMA eras (22). Together, these evolutions further support the need for continuous re-evaluation and adaptation of predictive tools in line with contemporary practice.

Despite its appealing findings, our study has some limitations. Its retrospective design may have introduced selection or information bias. Despite efforts to standardize imaging, biopsy, and pathological assessment across centres, inter-observer variability may have influenced the results. The relatively small sample size in Centre C, along with its low LNI prevalence, may limit the robustness of findings in that specific setting. Nonetheless, the multicentre nature of this study, the consistency of the methodological approach, and the inclusion of calibration and clinical utility analyses strengthen relevance of our conclusions.

Beyond the present findings, improving the robustness and generalizability of predictive models will require access to large, heterogeneous, and well-annotated datasets. Many existing nomograms have been developed from relatively small cohorts originating from high-volume academic centres, which may introduce institutional biases related to patient selection and clinical practice patterns. Expanding multicentre and international data collection initiatives is therefore critical to enhance calibration and external validity. In this context, the emergence of artificial intelligence (AI) and automated data capture systems in healthcare may play a pivotal role. AI-enabled platforms, particularly those integrated into digitally connected surgical environments, have the potential to systematically capture high-resolution clinical and intraoperative data at scale, facilitating large epidemiological analyses and the development of more robust and generalizable predictive models (23).

## CONCLUSIONS

In conclusion, while the Briganti nomogram remains the most validated tool available for LNI prediction in the era of MRI-targeted biopsies, its performance varies between centres. Our multicentre study is a call for urologist to locally validate and personalize their threshold selection. As new predictive algorithms (particularly those integrating PSMA-PET) emerge, future decision-making will likely become even more tailored, precise, and patient-centred. The integration of AI-driven data ecosystems and large-scale real-world datasets may further enhance the development and continuous recalibration of predictive models, paving the way toward more adaptive and generalizable decision-support tools.

## ADVANCING PRACTICE

This study highlights the importance of validating the 2019 Briganti nomogram within each clinical setting. Our results support individualized threshold selection for lymph node dissection, based on local performance and patient population characteristics. Fixed thresholds may lead to inconsistent or suboptimal treatment decisions, reinforcing the need for precision-guided surgical planning.

## Data Availability

All data generated or analysed during this study are included in this published article

## References

[1] Porcaro AB, Panunzio A, Orlando R, Montanaro F, Baielli A, Artoni F (2024). The 2012 Briganti nomogram not only predicts lymph node involvement but also disease progression in surgically treated intermediate-risk prostate cancer patients with PSA <10 ng/mL, ISUP grade group 3, and clinical stage up to cT2b. Int Braz J Urol.

[2] Kaneko M, Fujihara A, Iwata T, Ramacciotti LS, Palmer SL, Oishi M (2024). A nomogram to predict the absence of clinically significant prostate cancer in males with negative MRI. Int Braz J Urol.

[3] Morote J, Paesano N, Picola N, Muñoz-Rodriguez J, Ruiz-Plazas X, Muñoz-Rivero MV (2024). Validation of the Barcelona-MRI predictive model when PI-RADS v2.1 is used with transperineal prostate biopsies. Int Braz J Urol.

[4] Anger CM, Stallworth JL, Rais-Bahrami S (2024). Integrating risk calculators into routine clinical workflow for the detection of prostate cancer: next steps to achieve widespread adoption. Prostate Cancer Prostatic Dis.

[5] Denijs FB, van Harten MJ, Meenderink JJL, Leenen RCA, Remmers S, Venderbos LDF (2024). Risk calculators for the detection of prostate cancer: a systematic review. Prostate Cancer Prostatic Dis.

[6] Gandaglia G, Ploussard G, Valerio M, Mattei A, Fiori C, Fossati N (2019). A novel nomogram to identify candidates for extended pelvic lymph node dissection among patients with clinically localized prostate cancer diagnosed with magnetic resonance imaging-targeted and systematic biopsies. Eur Urol.

[7] Gandaglia G, Martini A, Ploussard G, Fossati N, Stabile A, De Visschere P (2020). External validation of the 2019 Briganti nomogram for the identification of prostate cancer patients who should be considered for an extended pelvic lymph node dissection. Eur Urol.

[8] Touijer KA, Sjoberg DD, Benfante N, Laudone VP, Ehdaie B, Eastham JA (2021). Limited versus extended pelvic lymph node dissection for prostate cancer: a randomized clinical trial. Eur Urol Oncol.

[9] Cornford P, van den Bergh RCN, Briers E, Van den Broeck T, Brunckhorst O, Darraugh J (2024). EAU-EANM-ESTRO-ESUR-ISUP-SIOG Guidelines on Prostate Cancer-2024 Update. Part I: Screening, Diagnosis, and Local Treatment with Curative Intent. Eur Urol.

[10] American College of Radiology; European Section of Uro-Radiology (2019). PI-RADS® Prostate Imaging — Reporting and Data System.

[11] van Leenders G (2020). The 2019 International Society of Urological Pathology (ISUP) Consensus Conference on grading of prostatic carcinoma. Am J Surg Pathol.

[12] Paner GP, Stadler WM, Hansel DE, Montironi R, Lin DW, Amin MB (2018). Updates in the eighth edition of the tumor-node-metastasis staging classification for urologic cancers. Eur Urol.

[13] Collins GS, Reitsma JB, Altman DG, Moons KGM (2015). Transparent reporting of a multivariable prediction model for individual prognosis or diagnosis (TRIPOD): the TRIPOD Statement. BMC Med.

[14] Collins GS, Dhiman P, Ma J, Schlussel MM, Archer L, Van Calster B (2024). Evaluation of clinical prediction models (part 1): from development to external validation. BMJ.

[15] Meijer D, van Leeuwen PJ, Roberts MJ, Siriwardana AR, Morton A, Yaxley JW (2021). External validation and addition of prostate-specific membrane antigen positron emission tomography to the most frequently used nomograms for the prediction of pelvic lymph-node metastases: an international multicenter study. Eur Urol.

[16] Vis AN, Meijer D, Roberts MJ, Siriwardana AR, Morton A, Yaxley JW (2023). Development and external validation of a novel nomogram to predict the probability of pelvic lymph-node metastases in prostate cancer patients using magnetic resonance imaging and molecular imaging with prostate-specific membrane antigen positron emission tomography. Eur Urol Oncol.

[17] Mazzone E, Gandaglia G, Robesti D, Rajwa P, Gomez Rivas J, Ibáñez L (2024). Which patients with prostate cancer and lymph node uptake at preoperative prostate-specific membrane antigen positron emission tomography/computerized tomography scan are at a higher risk of prostate-specific antigen persistence after radical prostatectomy? Identifying indicators of systemic disease by integrating clinical, magnetic resonance imaging, and functional imaging parameters. Eur Urol Oncol.

[18] Gandaglia G, Barletta F, Robesti D, Scuderi S, Rajwa P, Gomez Rivas J (2023). Identification of the optimal candidates for nodal staging with extended pelvic lymph node dissection among prostate cancer patients who underwent preoperative prostate-specific membrane antigen positron emission tomography. External validation of the Memorial Sloan Kettering Cancer Center and Briganti nomograms and development of a novel tool. Eur Urol Oncol.

[19] Gandaglia G, Barletta F, Scuderi S, Scilipoti P, Rajwa P, Huebner NA (2025). External validation of nomograms for the identification of pelvic nodal dissection candidates among prostate cancer patients with negative preoperative prostate-specific membrane antigen positron emission tomography. Eur Urol Oncol.

[20] Roberts MJ, Gandaglia G, Oprea-Lager DE, Stranne J, Cornford P, Tilki D (2025). Pelvic lymph node dissection in prostate cancer: evidence and implications. Eur Urol.

[21] Touijer KA, Vertosick EA, Sjoberg DD, Liso N, Nalavenkata S, Melao B (2025). Pelvic lymph node dissection in prostate cancer: update from a randomized clinical trial of limited versus extended dissection. Eur Urol.

[22] Kleiburg F, Dirix P, Fonteyne V, Bral S, De Troyer B, Sautois B (2025). Stage migration on prostate-specific membrane antigen positron emission tomography/computed tomography in comparison to conventional imaging in patients with high-risk prostate cancer referred for radiation therapy: results from the Phase 2/3 THUNDER Trial. Eur Urol Oncol.

[23] Reis LO, Moretti TBC (2026). Data-driven culture in medicine and surgery: policy pathways to learning health systems. Int J Gen Med.

