# Supplementary material for: Multicentre Validation of the 2019 Briganti Nomogram: One Threshold Does Not Fit All
Source: Int Braz J Urol. 2026 Apr 30;52(4):e20260023. doi: 10.1590/S1677-5538.IBJU.2026.0023 (PMC13400071; doi:10.1590/S1677-5538.IBJU.2026.0023)
Supplement: Supplementary Information [file 1677-6119-ibju-52-04-e20260023-suppl01.pdf]

## APPENDIX

**Supplementary Table 1 - Regression coefficients predicting lymph node involvement (LNI) from the 2019 Briganti nomogram as described by Gandaglia et al. Eur Urol 2019.**

| | Value |
|---|---|
| Intercept | -4.5974 |
| Preoperative PSA | 0.0416 |
| Extracapsular extension at MRI | 1.2214 |
| Seminal vesicle invasion at MRI | 1.4672 |
| Maximum lesion diameter at MRI | 0.0311 |
| Biopsy Gleason grade group 3 at targeted biopsy | 1.2032 |
| Biopsy Gleason grade group ≥ 4 at targeted biopsy | 1.8063 |
| Percentage of cores with clinically significant PCa at concomitant systematic biopsy | 0.0119 |

PSA = Prostate Specific Antigen; MRI = Magnetic Resonance Imaging; PCa = Prostate Cancer

**Supplementary Table 2 - Descriptives characteristic of the population for the development and validation datasets of the 2019-Briganti nomogram. Yr: Year; IQR: Inter-Quartile Range; DRE: Digital Rectal Examination; PI-RADS: Prostate Imaging – Reporting and Data System; MRI: Magnetic Resonance Imaging; ECE: Extra-Capsular Extension; SVI: Seminal Vesicle Invasion; ISUP: International Society of Urological Pathology.**

| | 2019 Briganti | | |
|---|---|---|---|
| | Development dataset | | **Validation dataset** (n=487) |
| | pN0 (n=433) | pN1 (n=62) | |
| **Age at surgery (yr), median (IQR)** | 65 (60-70) | 64 (60-71) | 65 (60-69) |
| **Preoperative PSA (ng/mL), median (IQR)** | 7.2 (5.1-11) | 11 (6.7-21) | 7.6 (5.5-10.8) |
| **Clinical stage at DRE, n (%)** | | | - |
| cT1 | 335 (77) | 30 (48) | |
| cT2 | 96 (22) | 21 (34) | |
| cT3 | 4 (1) | 11 (17) | |
| **PI-RADS score of index lesion, n (%)** | | | |
| No lesion | 0 (0) | 0 (0) | 0 (0) |
| 3 | 121 (28) | 4 (6) | 27 (5.5) |
| 4 | 235 (54) | 26 (42) | 268 (56) |
| 5 | 79 (18) | 32 (52) | 185 (39) |
| **Maximum lesion diameter of index lesion at MRI (mm), median (IQR)** | 10 (9-14) | 15 (10-18) | 11 (8-16) |
| **ECE at MRI, n (%)** | 49 (12) | 19 (31) | 79 (16) |
| **SVI at MRI, n (%)** | 13 (3) | 14 (22) | 20 (4.1) |
| **ISUP grade group on targeted biopsy, n (%)** | | | |
| Negative | 0 (0) | 0 (0) | 0 (0) |
| 1 | 72 (17) | 1 (2) | 72 (14) |
| 2 | 225 (52) | 15 (24) | 221 (45) |
| 3 | 72 (17) | 16 (26) | 124 (25) |
| 4 | 46 (11) | 17 (27) | 52 (11) |
| 5 | 20 (5) | 13 (21) | 18 (3.7) |
| **Number of cores taken at targeted biopsy, median (IQR)** | 3 (2-4) | 3 (2-4) | - |
| **Number of positive cores at targeted biopsy, median (IQR)** | 2 (1-3) | 2 (2-3) | - |
| **ISUP grade group on systematic biopsy, n (%)** | | | |
| Negative | 80 (18) | 4 (7) | 49 (10) |
| 1 | 100 (23) | 6 (10) | 70 (14) |
| 2 | 171 (40) | 14 (23) | 234 (48) |
| 3 | 44 (10) | 15 (24) | 69 (14) |
| 4 | 25 (6) | 9 (15) | 47 (10) |
| 5 | 15 (4) | 14 (23) | 18 (3.7) |

| | | | |
|---|---|---|---|
| **Number of cores taken at systematic biopsy, median (IQR)** | 12 (10-15) | 12 (10-16) | - |
| **Number of positive cores at systematic biopsy, median (IQR)** | 12 (0-37) * | 42 (17-76) * | - |
| **ISUP grade group (overall), n (%)** | | | - |
| 1 | 55 (13) | 1 (2) | |
| 2 | 236 (54) | 15 (24) | |
| 3 | 78 (18) | 16 (26) | |
| 4 | 45 (10) | 15 (24) | |
| 5 | 21 (5) | 15 (24) | |
| **ISUP grade group at final pathology, n (%)** | | | - |
| 1 | 15 (3.5) | 0 (0) | |
| 2 | 218 (50) | 3 (4.8) | |
| 3 | 147 (34) | 25 (40) | |
| 4 | 22 (5.1) | 4 (6.5) | |
| 5 | 30 (6.9) | 30 (48) | |
| **ECE at final pathology, n (%)** | 180 (41) | 20 (32) | 160 (33) |
| **SVI at final pathology, n (%)** | 40 (9.2) | 39 (63) | 61 (13) |
| **Positive surgical margin, n (%)** | 103 (24) | 40 (48) | - |
| **Lymph node invasion at final pathology, n (%)** | 0 (0) | 100 (100) | 38 (7.8) |

*Median cores with clinically significant prostate cancer on systematic biopsy, % (IQR)
